# Neonatal Necrotizing Enterocolitis

**DOI:** 10.1100/tsw.2011.69

**Published:** 2011-03-22

**Authors:** Saad Lahmiti, Abdelmounaim Aboussad

**Affiliations:** Neonatal Intensive Care Department, Mohammed VI University Hospital and Research Team for Childhood, Health and Development, Marrakech School of Medicine, Cadi Ayyad University, Marrakech, Morocco

**Keywords:** newborn, enterocolitis, preterm

## Abstract

Necrotizing enterocolitis (NEC) is a gastrointestinal disease that mostly affects premature infants. It involves infection and inflammation that causes destruction of the bowel. Although it affects only 1 in 2,000 to 4,000 births, or between 1 and 5% of neonatal intensive care unit admissions, NEC is the most common and serious gastrointestinal disorder among hospitalized preterm infants. We present a very representative abdominal X-ray of this disease.

A baby was born at home at 34 weeks of gestation, weighing 2100 g. On day 7, the baby developed a sepsis and was hospitalized in the neonatal intensive care unit. The clinical examination found an abdominal distention and an abdominal radiography (Fig. 1) showed typical radiological features of necrotizing enterocolitis (NEC). The plain abdominal film showed air in the bowel walls and a large pneumoperitoneum (subdiaphragmatic free air, perihepatic free air, double wall sign [a and b], triangle sign [c], and falciform ligament [d]). The infant was intubated, and was given inotropic support and antibiotics. Septic shock and disseminated intravascular coagulation soon developed that were unresponsive to treatment, and the infant died 12 h later.

NEC is an acquired gastrointestinal disease associated with significant morbidity and mortality in prematurely born neonates. NEC affects about 10% of premature infants <1500 g or 1–5% of all neonatal intensive care unit admissions. It characteristically strikes between 7 and 14 days of life, although increasingly, NEC has been documented several weeks after birth, particularly in very low-birth-weight infants[1]. With improving care at the end of the presurfactant era, the incidence of NEC declined briefly, but increased after surfactant use became a standard of care[2,3]. This reported increase is presumably because of the increased survival of extremely immature infants. Prematurity with its associated immature gastrointestinal host and blood flow regulation results in mucosal injury. Combined with enteral feeds and bacterial colonization, inflammatory mediators are released, leading to a propagated inflammatory response with both pro- and anti-inflammatory influences. If counter-regulatory responses to these inflammatory events are insufficient, pathologic changes to gut mucosa occur. In the preterm infant, the balance appears to favor proinflammatory influences resulting in NEC[4].

In the last decade, there has been a gradual realization that not all clinically diagnosed cases of NEC fit the classic pathological description[5]. For example, isolated small bowel perforation may be diagnosed as a form of NEC even though there is little bowel necrosis[5]. Furthermore, not all types of NEC carry the same morbidity or mortality risks.
